# Hypomethylation‐mediated activation of cancer/testis antigen KK‐LC‐1 facilitates hepatocellular carcinoma progression through activating the Notch1/Hes1 signalling

**DOI:** 10.1111/cpr.12581

**Published:** 2019-03-20

**Authors:** Zhiqiang Chen, Xueliang Zuo, Liyong Pu, Yao Zhang, Guoyong Han, Long Zhang, Zhengshan Wu, Wei You, Jianjie Qin, Xinzheng Dai, Hongbing Shen, Xuehao Wang, Jindao Wu

**Affiliations:** ^1^ Hepatobiliary Center, The First Affiliated Hospital of Nanjing Medical University, Key Laboratory of Liver Transplantation Chinese Academy of Medical Sciences, NHC Key Laboratory of Living Donor Liver Transplantation Nanjing China; ^2^ State Key Laboratory of Reproductive Medicine Nanjing Medical University Nanjing China; ^3^ Department of Gastrointestinal Surgery The First Affiliated Hospital, Yijishan Hospital of Wannan Medical College Wuhu China; ^4^ Department of Epidemiology and Biostatistics, Jiangsu Key Lab of Cancer Biomarkers, Prevention and Treatment, Collaborative Innovation Center for Cancer Personalized Medicine, School of Public Health Nanjing Medical University Nanjing China

**Keywords:** Cancer/testis antigen, epithelial‐mesenchymal transition, hepatocellular carcinoma, KK‐LC‐1, methylation

## Abstract

**Objectives:**

Kita‐Kyushu lung cancer antigen‐1 (KK‐LC‐1) is a cancer/testis antigen reactivated in several human malignancies. So far, the major focus of studies on KK‐LC‐1 has been on its potential as diagnostic biomarker and immunotherapy target. However, its biological functions and molecular mechanisms in cancer progression remain unknown.

**Materials and Methods:**

Expression of KK‐LC‐1 in HCC was analysed using RT‐qPCR, Western blot and immunohistochemistry. The roles of KK‐LC‐1 on HCC progression were examined by loss‐of‐function and gain‐of‐function approaches. Pathway inhibitor DAPT was employed to confirm the regulatory effect of KK‐LC‐1 on the downstream Notch signalling. The interaction of KK‐LC‐1 with presenilin‐1 was determined by co‐immunoprecipitation. The association of CpG island methylation status with KK‐LC‐1 reactivation was evaluated by methylation‐specific PCR, bisulphite sequencing PCR and 5‐Aza‐dC treatment.

**Results:**

We identified that HCC tissues exhibited increased levels of KK‐LC‐1. High KK‐LC‐1 level independently predicted poor survival outcome. KK‐LC‐1 promoted cell growth, migration, invasion and epithelial‐mesenchymal transition in vitro and in vivo. KK‐LC‐1 modulated the Notch1/Hes1 pathway to exacerbate HCC progression through physically interacting with presenilin‐1. Upregulation of KK‐LC‐1 in HCC was attributed to hypomethylated CpG islands.

**Conclusions:**

This study identified that hypomethylation‐induced KK‐LC‐1 overexpression played an important role in HCC progression and independently predicted poor survival. We defined the KK‐LC‐1/presenilin‐1/Notch1/Hes1 as a novel signalling pathway that was involved in the growth and metastasis of HCC.

## 1 INTRODUCTION

Hepatocellular carcinoma (HCC) is one of the most common and aggressive tumours worldwide.^1^ Although successful partial hepatectomy and liver transplantation have markedly improved survival, patients with HCC typically have poor prognosis. Elucidation of the molecular mechanisms underlying HCC progression is critical for identifying novel therapeutic targets and improving the prognosis of HCC patients.

Cancer/testis (CT) antigens are a well‐defined class of proteins that have been pursued as potential targets for anti‐cancer therapy. Cancer/testis antigens are typically restricted to gametes and trophoblasts in normal tissues but are aberrantly expressed in a range of human cancers.^2^, ^3^ Kita‐Kyushu lung cancer antigen‐1 (KK‐LC‐1), encoded by *CT83* or *Cxorf61 *gene, has been characterized as a CT antigen. KK‐LC‐1 is expressed in 40% of non‐small‐cell lung cancer tissues,^4^ 81.6% of gastric cancer^5^ and 75% of triple‐negative breast cancer.^6^ The expression rate of KK‐LC‐1 in human malignancies is markedly higher than the rates of other CT antigens. Correlated with *Helicobacter pylori* infection,^7^ KK‐LC‐1 can be frequently detected even in early stage of gastric cancer^8^ and at the pre‐cancerous condition of the stomach.^9^ KK‐LC‐1 is cancer cell selective and immunogenic, and expressed at high level and frequency in cancers,^6^ making it an ideal cancer immunotherapy target. A recent study has implicated the utility of KK‐LC‐1 as a target for photodynamic therapy in malignancies.^10^ However, the biological functions and underlying mechanisms of KK‐LC‐1 in human cancers remain obscure.

The Notch signalling is one of the well‐conserved pathways that are involved in multicellular organism development and homeostasis. Activation of Notch signalling helps to modulate fundamental intracellular processes, including cell cycle regulation, cell differentiation and apoptosis.^11^ Dysregulated Notch signalling could result in various diseases, such as leukaemia, Alagille syndrome, pulmonary arterial hypertension and ventricular septal defect.^12^ Perturbations of Notch signalling as well as its regulatory molecules have been observed in various cancers. In breast cancer, JAG1 activates Notch signalling and promotes tumour metastasis.^13^ Inhibition of Notch1/2 could suppress cell proliferation in renal cell carcinoma.^14^ Notch1 overexpression leads to enhanced tumour growth in melanoma.^15^ Notch signalling has been reported to be activated in HCC and promotes liver tumour formation.^16^ Upon ligand binding, Notch undergoes a series of cleavage events to generate its activated form NICD. γ‐Secretase mediates the last step of the cleavage and fine‐tunes the level of NICD in cytoplasm.^17^ As a catalytic subunit of γ‐secretase complex, presenilin‐1 contributes to the intricate regulation of the Notch signalling.^18^

A previous study mapped the landscape of CT genes in 19 cancer types^19^ and suggested *CT83*, the gene that encodes KK‐LC‐1 protein, as a CT gene overexpressed in liver cancer. Here, we reported that upregulated KK‐LC‐1 played a role in HCC malignant phenotype in vitro and in vivo. With hypomethylated CpG islands leading to its overexpression in HCC, KK‐LC‐1 modulated the presenilin‐1/Notch1/Hes1 signalling and controlled cell proliferation, invasiveness and epithelial‐mesenchymal transition (EMT). To our knowledge, we demonstrated for the first time that KK‐LC‐1 was involved in HCC progression and may serve as a prognostic predictor for HCC patients.

## 2 METHODS AND MATERIALS

### 2.1 Patient samples

We obtained paired HCC and adjacent non‐tumorous liver tissues from 60 patients who underwent surgical resection in the First Affiliated Hospital of Nanjing Medical University from January 2011 to December 2012. No chemotherapy, radiotherapy or targeted therapy was performed prior to surgery. The study was approved by the Ethic Committee of the First Affiliated Hospital of Nanjing Medical University. Written informed consent was obtained from all patients.

### 2.2 Cell lines and cell culture

Human hepatic cell line QSG‐7701 and HCC cell lines SMMC7721, HepG2, Hep3B, HLF, Focus and Huh7 were obtained from the Chinese Academy of Sciences Cell Bank (Shanghai, China). HCCLM3 cell line was acquired from Liver Cancer Institute, Zhongshan Hospital, Fudan University (Shanghai, China). Cells were cultured in Dulbecco's modified Eagle's medium (DMEM, Gibco, Thermo Fisher Scientific, Carlsbad, CA, USA) supplemented with 10% foetal bovine serum (FBS), 100 units/mL penicillin and 100 μg/mL streptomycin. All cells were maintained in a 37°C incubator with an atmosphere of 5% CO_2_.

### 2.3 RNA extraction and quantitative real‐time polymerase chain reaction (RT‐qPCR)

TRIzol reagent (Invitrogen, Carlsbad, CA, USA; Thermo Fisher Scientific) was used to extract total RNA from tissues and cells. cDNA synthesis was performed with PrimeScript RT Master Mix (TaKaRa, Dalian, China), and RT‐qPCR was performed using TB Green Premix Ex Taq (TaKaRa) on the Applied Biosystems 7900HT Fast Real‐Time PCR System (Applied Biosystems, Foster City, CA, USA; Thermo Fisher Scientific). GAPDH was used as the internal control. The 2^‐ΔΔCt^ method was employed to analyse the relative expression. The PCR primers used are presented in Table S1.

### 2.4 Western blot analysis and co‐immunoprecipitation

Western blot was performed using an SDS‐PAGE Electrophoresis System as described previously^20^ with antibodies specific for KK‐LC‐1 (Abcam, Cambridge, MA, USA), E‐cadherin (Cell Signaling Technology, Beverly, MA, USA), N‐cadherin (Cell Signaling Technology), vimentin (Cell Signaling Technology), Snail (Cell Signaling Technology), NICD1 (Cell Signaling Technology), Hes1 (Cell Signaling Technology), presenilin‐1 (Abcam) and GAPDH (Cell Signaling Technology). Signals were detected using enhanced chemiluminescence reagents (Thermo Fisher Scientific). The total proteins were extracted from cells for co‐immunoprecipitation using protein A/G‐agarose beads (Santa Cruz Biotechnology, Santa Cruz, CA, USA) according to the manufacturer's instructions. Antibodies against KK‐LC‐1 (FabGennix, Frisco, TX, USA) and presenilin‐1 (Abcam) were used for co‐immunoprecipitation. The bound proteins were analysed by Western blot.

### 2.5 Immunohistochemistry analysis and immunofluorescence

Expression of KK‐LC‐1, PCNA, E‐cadherin, vimentin and NICD1 was evaluated using an immunohistochemical method described previously.^20^ The intensity of immunohistochemical staining was set as 0 for negative, 1 for weak, 2 for medium and 3 for strong. The percentage of stained area was scored as 0 for 0%‐10%, 1 for 11%‐25%, 2 for 26%‐50%, 3 for 51%‐75% and 4 for 76%‐100%. The overall staining score was calculated by multiplying the intensity score and staining area score as previously described.^20^ Immunofluorescence was carried out as previously reported.^20^

### 2.6 Construction of stable cell lines

To observe the knockdown effects of KK‐LC‐1, HCCLM3 and SMMC7721 cells were transfected with the shRNA‐KK‐LC‐1 (sh‐KK‐LC‐1) or control (sh‐con) viruses (GenePharma, Shanghai, China). To obtain cell lines stably overexpressing KK‐LC‐1, Focus and Huh7 cells were infected with the Lenti‐KK‐LC‐1 (KK‐LC‐1‐OE) or empty vector control (Lv‐con) viruses (GenePharma). The infection efficiency was confirmed by RT‐qPCR and Western blot. The shRNA used for targeting presenilin‐1 was purchased from GenePharma. The sequences for sh‐KK‐LC‐1 and sh‐presenilin‐1 are listed in Table S1.

### 2.7 Cell proliferation assays

Cell Counting Kit‐8 (CCK‐8), 5‐ethynyl‐2’‐deoxyuridine (EdU) incorporation assay and colony formation assay were carried out as described in previous study.^21^

### 2.8 Cell migration and invasion assays

Cell migration and invasion were evaluated with transwell inserts coated without Matrigel (for the migration assay) or with Matrigel (for the invasion assay) as reported previously.^21^

### 2.9 Animal studies

All animal studies were approved by the Institutional Animal Care and Use Committee of Nanjing Medical University. Four‐week‐old male nude mice were purchased from the Animal Core Facility of Nanjing Medical University (Nanjing, China). For in vivo tumour growth assays, cells (5 × 10^6^) were injected subcutaneously with 0.1 mL of the suspension into the flanks. Tumour growth was measured every three days with a calliper. The mice were sacrificed after four weeks, and the xenografts were dissected and weighed. The tumour volume was calculated according to the formula: V = L × W^2^ × 0.5 (V, volume; L, length; W, width). Xenografts were used for further Western blot and immunohistochemistry analysis. For survival analysis, we constructed the xenograft mice model using eight mice per group and observed the overall survival with 12 weeks as the cut‐off. For in vivo tumour metastasis assays, 2 × 10^6^ cells were injected into nude mice through the tail vein. Six weeks later, all mice were sacrificed to observe the tumour metastasis in liver and lung. The metastatic tissues were photographed, and the lung was further analysed by haematoxylin and eosin (HE) staining.

### 2.10 Methylation‐specific PCR (MSP)

Genomic DNA from HCC tumour tissues and noncancerous liver tissues was extracted using the TIANamp Genomic DNA Kit (TIANGEN Biotech, Beijing, China) according to the manufacturer's instructions. DNA was subjected to sodium bisulphite treatment using the Methylation‐Gold Kit (Zymo Research Corporation, Irvine, CA, USA). Forward and reverse primers for the methylated sequence (M) and the unmethylated sequence (U) are listed in Table S1.

### 2.11 Bisulphite sequencing PCR (BSP)

Genomic DNA was treated with bisulphite using the Methylation‐Gold Kit (Zymo Research Corporation) according to the manufacturer's instructions. We predicted CpG island of the *CT83* gene locus (between −2000 and +400 bp from transcription start site, NC_000023.11: c116465033‐116462634) by MethPrimer^22^ (http://www.urogene.org/methprimer/) and designed primers for quantitative methylation analysis. Sequences of primers are listed in Table S1. The coverage of product of the primer was between −90 and +250 bp (NC_000023.11: c116463123‐116462783) from transcription start site of the *CT83* gene and contained 17 CpG sequences. The modified DNA was amplified using the specific sequencing PCR primers. The 341‐bp PCR products were cloned into pMD.19‐T Vector (TaKaRa). At least ten independent colonies of each sample were sequenced by GeneCreate (Wuhan, China).

### 2.12 Statistical analysis

Statistical analysis was performed using the SPSS 21.0 (IBM Corporation, Armonk, NY, USA) and GraphPad Prism 6 (GraphPad Software, La Jolla, CA, USA). Wilcoxon signed‐rank test was used to compare the expression level of KK‐LC‐1 between HCC samples and adjacent non‐tumorous liver specimens. Other quantitative variables were assessed by Student's *t* test. Correlations between KK‐LC‐1 and clinicopathologic characteristics of HCC patients were analysed using Fisher's exact test. Spearman's rank correlation coefficient was used to demonstrate the relationship between *CT83 *DNA methylation level and expression level of *CT83*. Patient survival was compared with the Kaplan‐Meier method, and the significance was determined by the log‐rank test. Cox proportional hazards regression model was used for multivariate survival analysis. Values are expressed as mean ± standard error of the mean. Statistical significance was concluded at *P < 0.05, **P < 0.01, ***P < 0.001.

## 3 RESULTS

### 3.1 KK‐LC‐1 is frequently overexpressed in HCC and serves as a prognostic indicator

**Figure 1:**
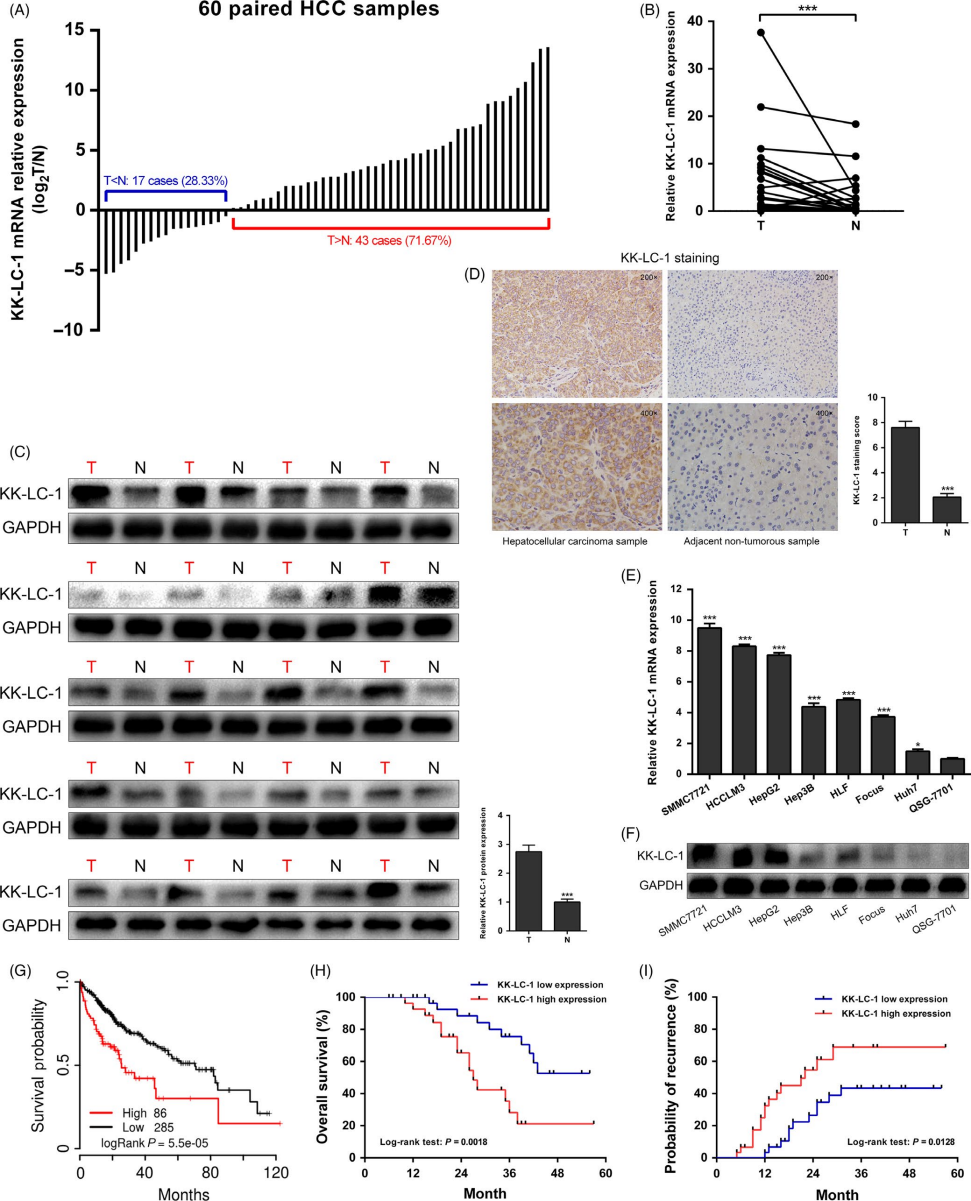
KK‐LC‐1 is frequently overexpressed in HCC and serves as a prognostic indicator. A, The expression level of KK‐LC‐1 mRNA was compared between 60 paired HCC (T) and adjacent non‐tumorous (N) tissues using RT‐qPCR. GAPDH was used for normalization. B, KK‐LC‐1 mRNA expression level was elevated in 60 paired HCC tissues compared to that in adjacent non‐tumorous samples. Wilcoxon signed‐rank test was adopted to analyse the difference between the groups. The data are shown as the mean ± SE. ***P < 0.001. C, Protein expression levels of KK‐LC‐1 in 20 matched HCC tissues and adjacent non‐tumorous samples. The data are shown as the mean ± SE. ***P < 0.001. D, Representative images of immunohistochemical staining of KK‐LC‐1 in paired HCC tissues. Quantitation of the immunohistochemical staining score was shown. The data are presented as the mean ± SE. ***P < 0.001. E, Expression levels of KK‐LC‐1 mRNA in seven HCC cell lines (SMMC7721, HCCLM3, HepG2, Hep3B, HLF, Focus and Huh7) and human normal hepatic cell line QSG‐7701 were evaluated using RT‐qPCR. The data are shown as the mean ± SE. *P < 0.05, ***P < 0.001. F, Protein expression levels of KK‐LC‐1 in HCC cell lines and human normal hepatic cell line. G, TCGA survival data were retrieved using TCGAportal and analysed to generate the Kaplan‐Meier curve of overall survival among 371 HCC patients stratified by KK‐LC‐1 expression level according to the median value. Patients with higher KK‐LC‐1 level had poorer survival than those with lower KK‐LC‐1 level (*P* = 5.5 × 10^−5^). H, Kaplan‐Meier curve of overall survival among the 60‐patient HCC cohort stratified by KK‐LC‐1 expression. Patients with higher KK‐LC‐1 expression presented worse overall survival (*P* = 0.0018). I, Probability of recurrence was evaluated by Kaplan‐Meier curve using the 60‐patient HCC cohort. Patients with high KK‐LC‐1 level exhibited high probability of recurrence (*P* = 0.0128).

To understand the role of KK‐LC‐1 in liver cancer, we first determined the expression levels of KK‐LC‐1 in 60 pairs of HCC samples and adjacent non‐tumorous liver specimens by RT‐qPCR. We observed that KK‐LC‐1 was frequently elevated in HCC tissues compared with that in the matched non‐tumorous liver samples (Figure 1A,B). Western blot analysis confirmed the upregulated protein levels of KK‐LC‐1 in most HCC cases (Figure 1C). We also performed immunohistochemical analysis of KK‐LC‐1 expression, and an increased amount of KK‐LC‐1 was noticed in HCC specimens as compared with the adjacent non‐tumorous samples (Figure 1D). In addition to its expression in tissue samples, we identified the mRNA and protein expression levels of KK‐LC‐1 in a number of HCC cell lines. KK‐LC‐1 exhibited significant differential expression in HCC cell lines compared to human normal hepatic cell line (Figure 1E, F).

Next, we investigated the clinical significance of KK‐LC‐1 expression in HCC patients. Based on the median value of KK‐LC‐1 mRNA level (2^‐ΔΔCt^ = 5.52375 × 10^−6^), all 60 patients were divided into high KK‐LC‐1 level group and low KK‐LC‐1 expression group. As shown in Table S2, KK‐LC‐1 upregulation was significantly correlated with multiple malignant clinicopathological characteristics of HCC, including α‐fetoprotein (*P = *0.017), Edmondson‐Steiner grading (*P* = 0.035), microvascular invasion (*P* = 0.013) and TNM stage (*P* = 0.003). To determine the prognostic value of KK‐LC‐1 in HCC, we used TCGAportal (http://tumorsurvival.org/index.html) to analyse the survival data from TCGA. A total of 371 HCC patients from the TCGA database were separated into high KK‐LC‐1 expression group and low KK‐LC‐1 expression group according to the median level of KK‐LC‐1 expression. Kaplan‐Meier analysis using TCGA data suggested that patients with higher KK‐LC‐1 expression exhibited much worse overall survival (Figure 1G; *P* = 5.5 × 10^‐5^). In addition to the TCGA data, we conducted survival analysis on the recruited 60 HCC patients from our centre. Our data suggested that KK‐LC‐1 overexpression correlated with poor overall survival (Figure 1H; *P* = 0.0018) and high probability of recurrence (Figure 1I; *P* = 0.0128). Cox proportional hazards regression model was employed to perform the multivariate survival analysis on the 60‐patient cohort. As shown in Table S3, the results showed that patients with high KK‐LC‐1 expression were at high risk of poor overall survival [hazard ratio (HR) 5.912, 95% confidence interval (CI): 2.307‐15.147, *P* < 0.001] and high probability of recurrence (HR 2.787, 95% CI: 1.232‐6.305, *P* = 0.014). Collectively, these data indicated that KK‐LC‐1 was frequently overexpressed in HCC and associated with HCC clinicopathological characteristics. KK‐LC‐1 may be used as a potential prognostic indicator for HCC.

### 3.2 KK‐LC‐1 is required for the maintenance of malignant phenotypes of HCC cells

**Figure 2:**
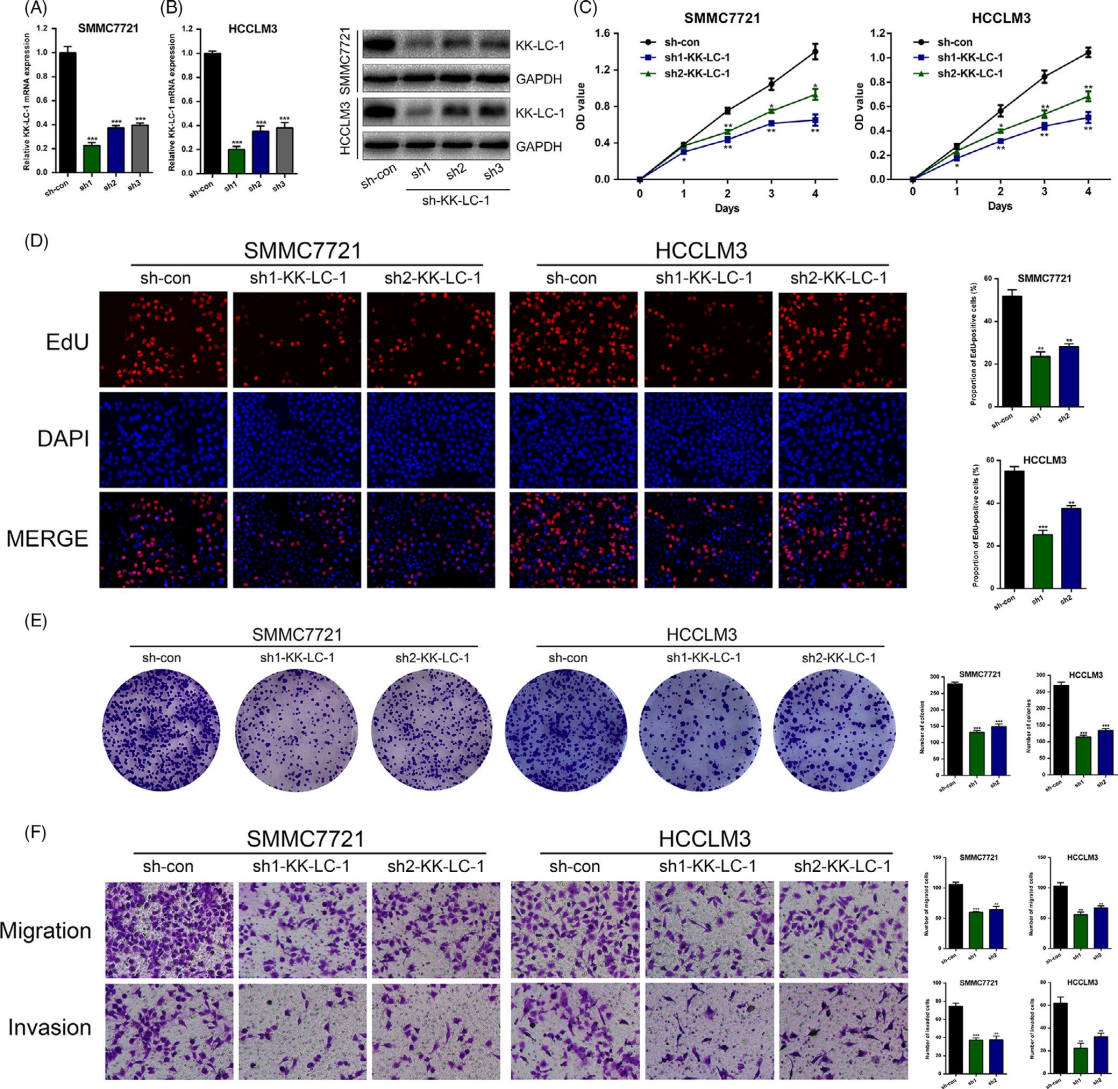
KK‐LC‐1 knockdown suppresses HCC cell proliferation, migration and invasion in vitro. A, KK‐LC‐1 expression in SMMC7721 and HCCLM3 was stably knocked down using shRNA. RT‐qPCR was performed to assess the knockdown efficiency. B, Western blot was conducted to evaluate the effects of sh‐KK‐LC‐1 on SMMC7721 and HCCLM3 cells. C, CCK‐8 assays were used to analyse cell proliferation in HCC cells with KK‐LC‐1 knockdown. D, EdU assays were performed to examine the effects of KK‐LC‐1 knockdown on HCC cell proliferation ability. Proportion of EdU‐positive cells was determined and statistically analysed. E, Colony formation assays were conducted in sh‐KK‐LC‐1 HCC cells and control cells. Number of colonies was counted and shown as histogram. F, Transwell assays were used to analyse HCC cell motility. Migrated and invaded cells were quantified and statistically analysed. The data are shown as the mean ± SE. *P < 0.05, **P < 0.01, ***P < 0.001.

**Figure 3:**
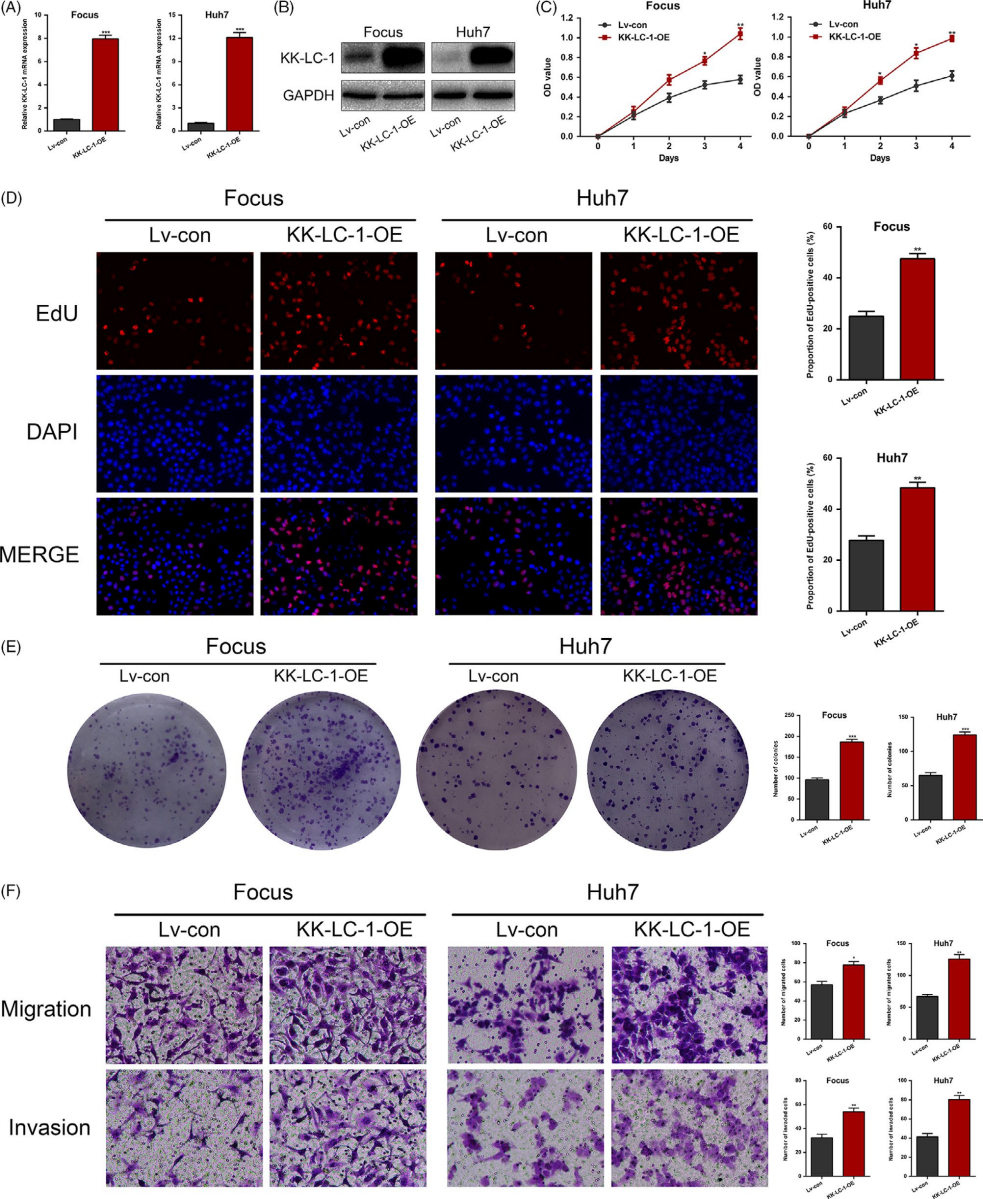
Overexpression of KK‐LC‐1 promotes HCC cell proliferation, migration and invasion in vitro. A, Lentiviruses were used to stably upregulate KK‐LC‐1 expression in Focus and Huh7 cells, named as KK‐LC‐1‐OE cells. RT‐qPCR was performed to assess the KK‐LC‐1 mRNA level in KK‐LC‐1‐OE Focus and Huh7 cells. B, Western blot was conducted to confirm the elevated KK‐LC‐1 protein level in KK‐LC‐1‐OE Focus and Huh7 cells. C, CCK‐8 assays were used to analyse cell proliferation in HCC cells with KK‐LC‐1 overexpression. D, EdU assays were performed to examine the effects of KK‐LC‐1 overexpression on HCC cell proliferation ability. Proportion of EdU‐positive cells was determined and statistically analysed. E, Colony formation assays were conducted in KK‐LC‐1‐OE HCC cells and controls. Number of colonies was counted and shown as histogram. F, Transwell assays were used to analyse HCC cell motility. Migrated and invaded cells were quantified and statistically analysed. The data are shown as the mean ± SE. *P < 0.05, **P < 0.01, ***P < 0.001.

To understand the functional role of KK‐LC‐1 in HCC cells, we first performed knockdown of KK‐LC‐1 via shRNA in SMMC7721 and HCCLM3 with high levels of KK‐LC‐1. Three shRNAs (sh1‐KK‐LC‐1, sh2‐KK‐LC‐1 and sh3‐KK‐LC‐1) were designed to knock down KK‐LC‐1 expression. Expression level of KK‐LC‐1 was determined by RT‐qPCR and Western blot; sh1‐KK‐LC‐1 was the most effective one (Figure 2A,B). Meanwhile, cells with stable lentiviral KK‐LC‐1 overexpression in Focus and Huh7 were also constructed and named as KK‐LC‐1‐OE cells subsequently (Figure 3A,B). We then performed a variety of in vitro assays to evaluate the effects of KK‐LC‐1 knockdown or overexpression on cell functions, including proliferation, migration and invasion. Using CCK‐8, EdU and colony formation assays, we observed a dramatic reduction in the proliferative capacity of sh‐KK‐LC‐1 SMMC7721 and HCCLM3 cells (Figure 2C‐E). On the contrary, overexpression of KK‐LC‐1 in Focus and Huh7 cells exhibited a significant increase in proliferative capacity (Figure 3C‐E). Additionally, transwell assays were performed, and we found that KK‐LC‐1 promoted HCC cell migration and invasion (Figure 2F and Figure 3F).

### 3.3 KK‐LC‐1 is associated with EMT process in HCC

**Figure 4:**
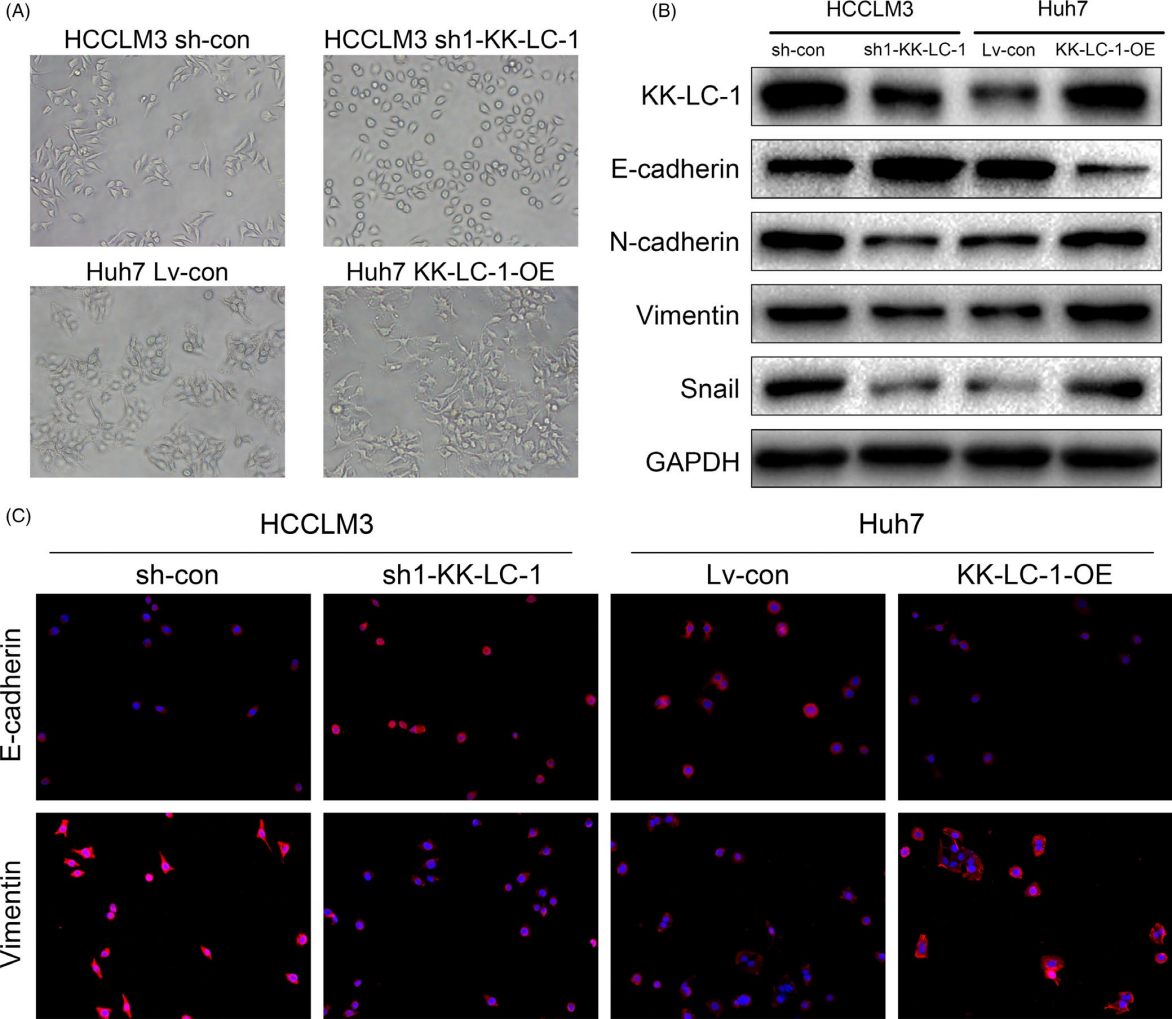
KK‐LC‐1 is associated with EMT process in HCC. A, Representative images of cellular morphology of sh1‐KK‐LC, KK‐LC‐OE and their control cells. B, Expression levels of EMT markers mediated by KK‐LC‐1 were detected by Western blot. C, Immunofluorescence for E‐cadherin and vimentin showed that KK‐LC‐1 affected expressions of EMT markers. Representative images of sh1‐KK‐LC, KK‐LC‐OE and their control cells are shown

Previous reports have indicated that a number of CT antigens induce EMT in human malignancies.^23^ Therefore, we speculated that KK‐LC‐1 might be involved in the EMT process. sh‐KK‐LC‐1 HCCLM3 cells exhibited cobblestone‐like epithelial morphology, whereas the control HCCLM3 cells mostly looked like mesenchymal spindle in appearance. Inversely, KK‐LC‐1‐OE Huh7 cells changed to a mesenchymal phenotype as compared with epithelial‐like control Huh7 cells (Figure 4A). KK‐LC‐1 knockdown led to elevated E‐cadherin and decreased N‐cadherin, vimentin and Snail at protein levels (Figure 4B). Consistently, immunofluorescence analysis indicated that KK‐LC‐1 knockdown increased the expression of E‐cadherin and reduced vimentin expression in sh‐KK‐LC‐1 HCCLM3 cells (Figure 4C). With regard to KK‐LC‐1‐OE Huh7 cells, we observed the opposite changes of EMT markers using Western blot and immunofluorescence analysis (Figure 4B,C). These results indicated that KK‐LC‐1 was associated with EMT in HCC.

### 3.4 KK‐LC‐1 promotes HCC growth and metastasis in vivo

**Figure 5:**
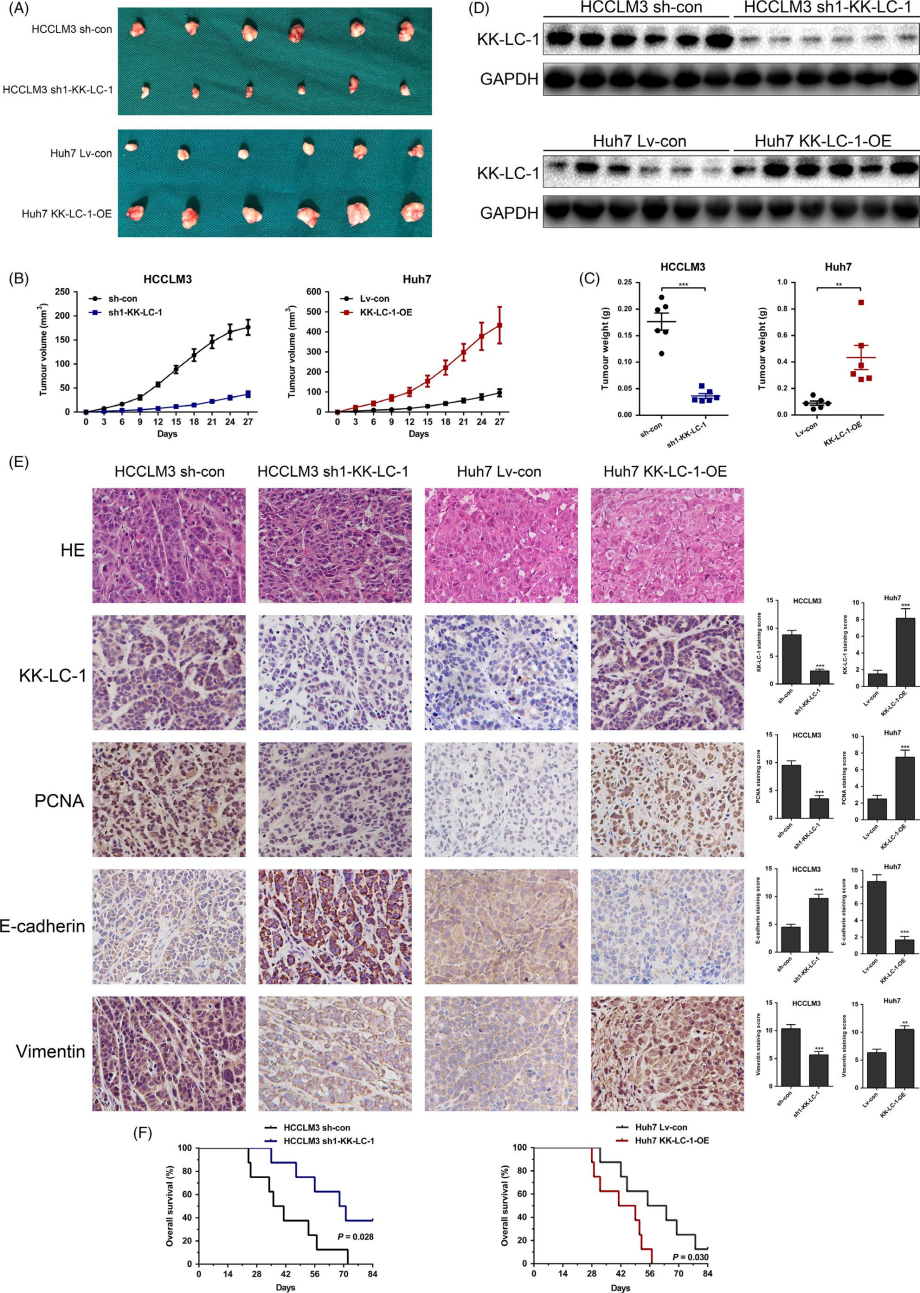
KK‐LC‐1 promotes HCC growth in vivo. A, HCCLM3 sh1‐KK‐LC‐1 and Huh7 KK‐LC‐1‐OE cells were injected into nude mice as indicated. After four weeks, the mice were sacrificed to harvest the tumours. B, The tumour growth curve was plotted in the sh1‐KK‐LC‐1 group, the KK‐LC‐1‐OE group and their controls. The data are shown as the mean ± SE. C, The tumour weight was measured in the sh1‐KK‐LC‐1 group, the KK‐LC‐1‐OE group and their controls. The data are shown as the mean ± SE. **P < 0.01, ***P < 0.001. D, Western blot analysis of the xenografts showed the consistent knockdown or overexpression of KK‐LC‐1 in xenograft mice model. E, HE, KK‐LC‐1, PCNA, E‐cadherin and vimentin immunostaining of the tumours derived from xenograft mice model. F, Kaplan‐Meier curves of the sh1‐KK‐LC‐1 group, the KK‐LC‐1‐OE group and their controls. Mice injected with HCCLM3 sh1‐KK‐LC‐1 cells had longer overall survival (*P* = 0.028), whereas mice in the Huh7 KK‐LC‐1‐OE group presented worse survival outcome (*P* = 0.030).

**Figure 6:**
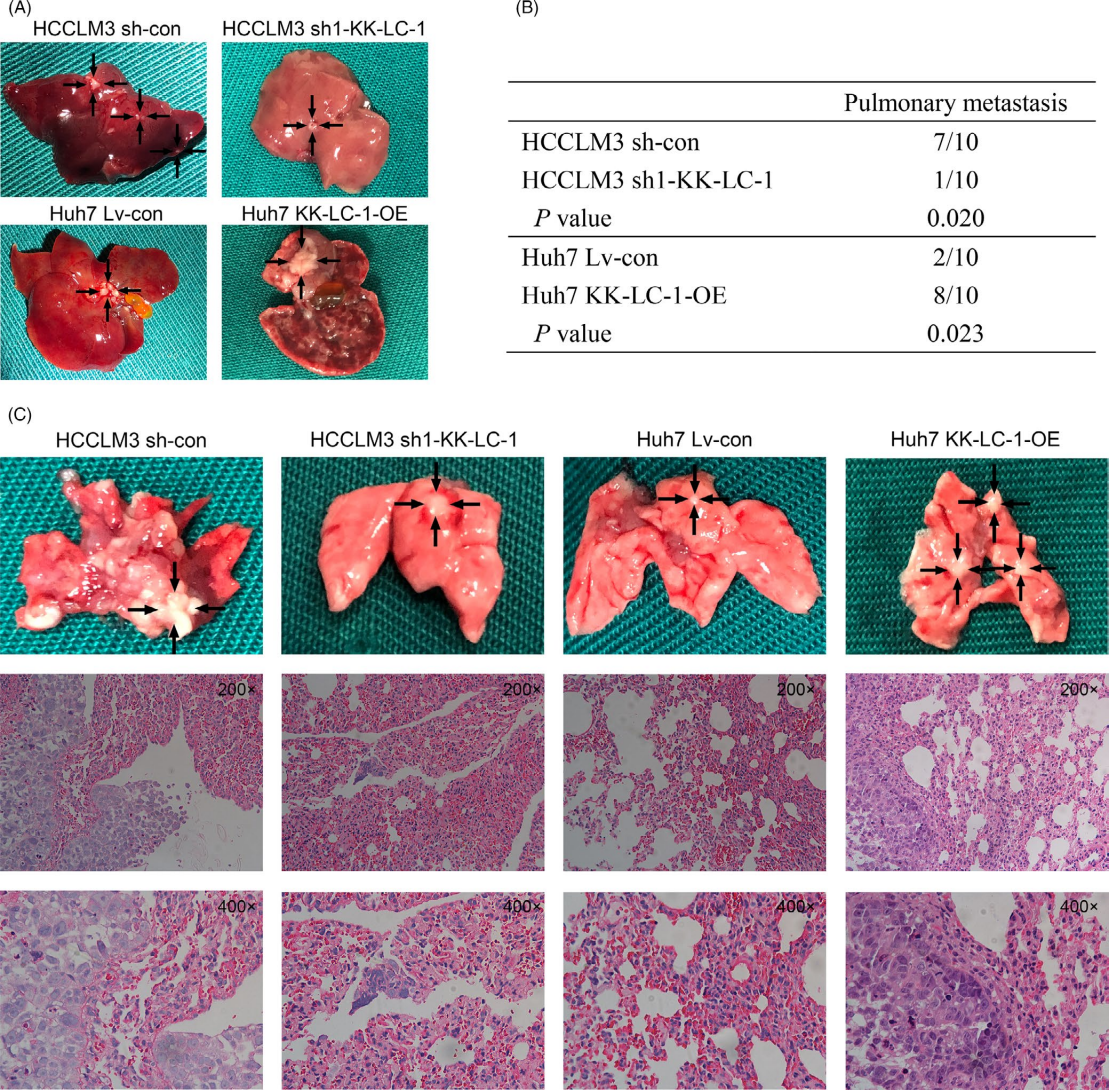
KK‐LC‐1 promotes HCC growth and metastasis in vivo. A, Representative images of the liver metastatic nodules in the sh1‐KK‐LC‐1 group, the KK‐LC‐1‐OE group and their controls. B, The numbers of the mice with lung metastases in the sh1‐KK‐LC‐1 group, the KK‐LC‐1‐OE group and their controls. C, Representative images of the lung metastatic nodules and HE staining to reveal metastatic tumour cells in the sh1‐KK‐LC‐1 group, the KK‐LC‐1‐OE group and their controls

To explore the effects of KK‐LC‐1 on HCC in vivo growth, we established a xenograft mice model to test HCC cell proliferation. HCC cells with KK‐LC‐1 knockdown or overexpression were inoculated subcutaneously into nude mice. Our results showed that the growth of tumours from sh‐KK‐LC‐1 cells was significantly inhibited compared with that of the control xenografts. In contrast, the growth of tumours from KK‐LC‐1‐OE cells was markedly accelerated compared with that of the controls (Figure 5A‐C). Western blot and immunohistochemical analysis of the xenografts showed that KK‐LC‐1 knockdown or overexpression was maintained in the xenografts (Figure 5D,E). PCNA staining of the xenografts indicated lower proliferation rate in mice inoculated with sh‐KK‐LC‐1 HCCLM3 cells and higher proliferation rate in mice injected with KK‐LC‐1‐OE Huh7 cells (Figure 5E). Wondering whether KK‐LC‐1 was associated with EMT markers in HCC in vivo, we performed E‐cadherin and vimentin immunostaining analysis on tumours derived from xenograft mice models. As indicated in Figure 5E, we observed higher expression of E‐cadherin and lower expression of vimentin in xenografts derived from sh‐KK‐LC‐1 HCCLM3 cells, and lower E‐cadherin and higher vimentin in tumours originating from KK‐LC‐1‐OE Huh7 cells. Survival analysis indicated that the sh‐KK‐LC‐1 HCCLM3 group presented longer overall survival, whereas mice inoculated with KK‐LC‐1‐OE Huh7 cells had poorer survival (Figure 5F). Given that EMT is an early phenotype contributing to tumour metastasis, we next constructed tail vein injection mice model to evaluate the effect of KK‐LC‐1 on HCC metastasis. In this model system, the incidence of both intrahepatic and pulmonary metastases in the sh‐KK‐LC‐1 group was significantly decreased compared with the control group, whereas the KK‐LC‐1‐OE group exhibited higher intrahepatic and pulmonary metastatic capacity compared with the control group (Figure 6A‐C).

### 3.5 KK‐LC‐1 activates the Notch1 signalling in HCC

**Figure 7:**
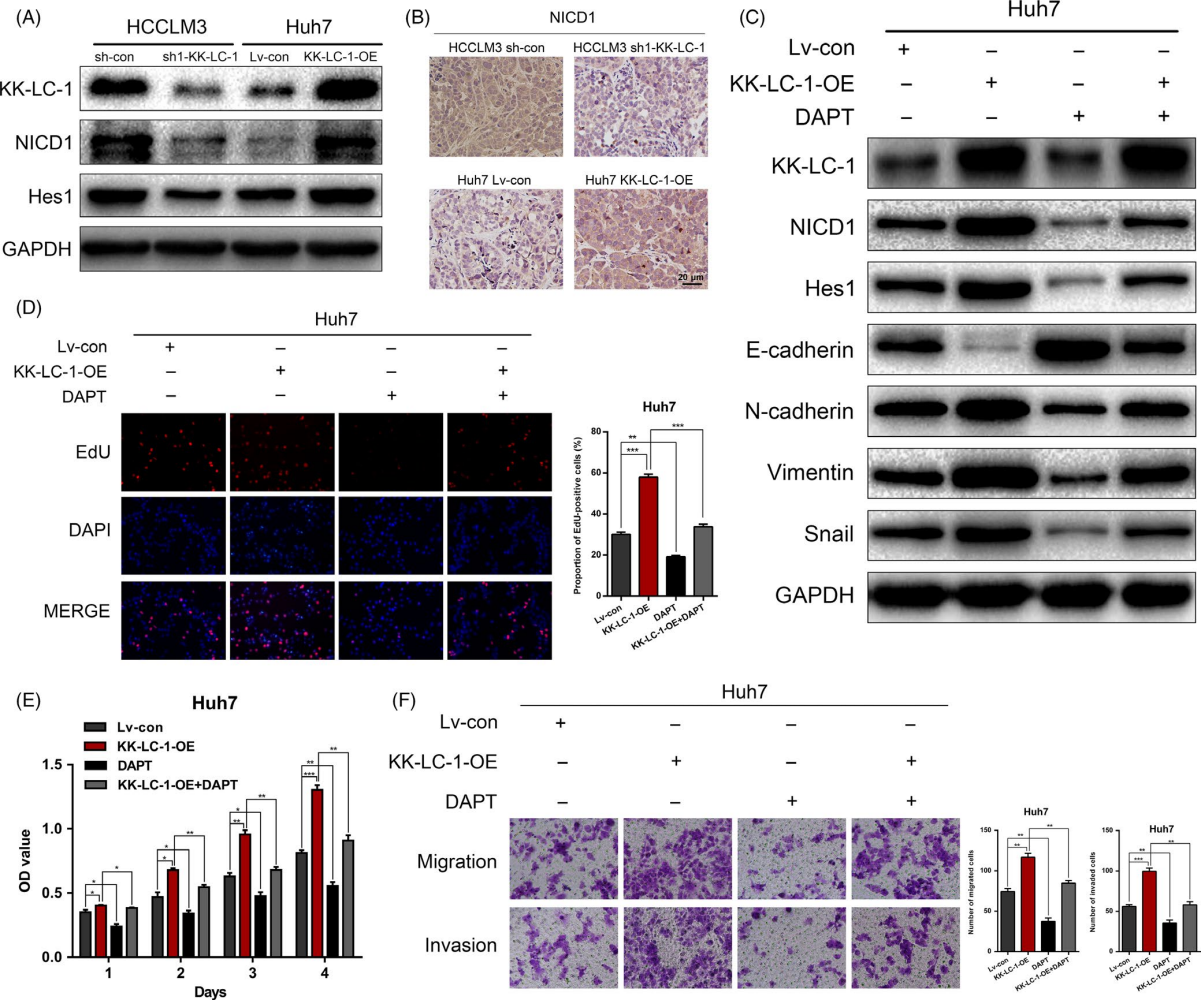
KK‐LC‐1 activates the Notch1 signalling in HCC. A, Protein expressions of KK‐LC‐1, NICD1 and Hes1 were detected in HCCLM3 sh1‐KK‐LC‐1, Huh7 KK‐LC‐1‐OE and their control cells. B, Representative images of NICD1 immunostaining of the tumours derived from xenograft mice model. C, Western blot was performed to evaluate the expressions of NICD1, Hes1 and EMT markers in Huh7 KK‐LC‐1‐OE cells treated with DAPT. D, EdU assays were conducted to assess the proliferation ability of Huh7 KK‐LC‐1‐OE cells treated with DAPT. Proportion of EdU‐positive cells was determined and statistically analysed. E, CCK‐8 assays for Huh7 KK‐LC‐1‐OE cells treated with DAPT. F, Transwell assays were used to analyse HCC cell migration and invasion in Huh7 KK‐LC‐1‐OE cells treated with DAPT. Migrated and invaded cells were quantified and statistically analysed. The data are shown as the mean ± SE. *P < 0.05, **P < 0.01.

Identification of the downstream signalling mediators of KK‐LC‐1 is crucial for understanding the mechanisms underlying KK‐LC‐1 regulation of HCC cells. Previous reports have indicated the significant role of Notch1 signalling in hepatic oncogenesis.^24^, ^25^ Recent work has also implicated the activation of Notch1 signalling in CT antigen‐mediated cancer progression.^28^ As shown in Figure 7A, KK‐LC‐1 knockdown greatly attenuated the expression of NICD1 and Notch1 effector Hes1 in HCCLM3 cells, and KK‐LC‐1 overexpression significantly enhanced the activity of Notch1 signalling in Huh7 cells. Immunohistochemical analysis of the xenografts indicated the same correlation between KK‐LC‐1 and NICD1 in vivo (Figure 7B). To confirm the activation of Notch1 signalling by KK‐LC‐1 in liver cancer, we treated Huh7 cells with 5 μmol/L of N‐[N‐(3,5‐difluorophenacetyl)‐L‐alanyl]‐S‐phenylglycine t‐butylester (DAPT; Selleck, Houston, TX, USA) for three days to suppress the release of NICD1. Western blot analysis showed that DAPT treatment inhibited KK‐LC‐1‐induced NICD1 release and Hes1 expression, and reversed the altered levels of EMT markers in KK‐LC‐1‐OE Huh7 cells (Figure 7C). Rescue experiments also showed that DAPT treatment reduced the elevated proliferative, migrative and invasive ability of KK‐LC‐1‐OE Huh7 cells (Figure 7D‐F). These results concluded that KK‐LC‐1 facilitated HCC progression through activating Notch1 signalling.

### 3.6 KK‐LC‐1 physically interacts with presenilin‐1 to regulate the Notch1 signalling in HCC

**Figure 8:**
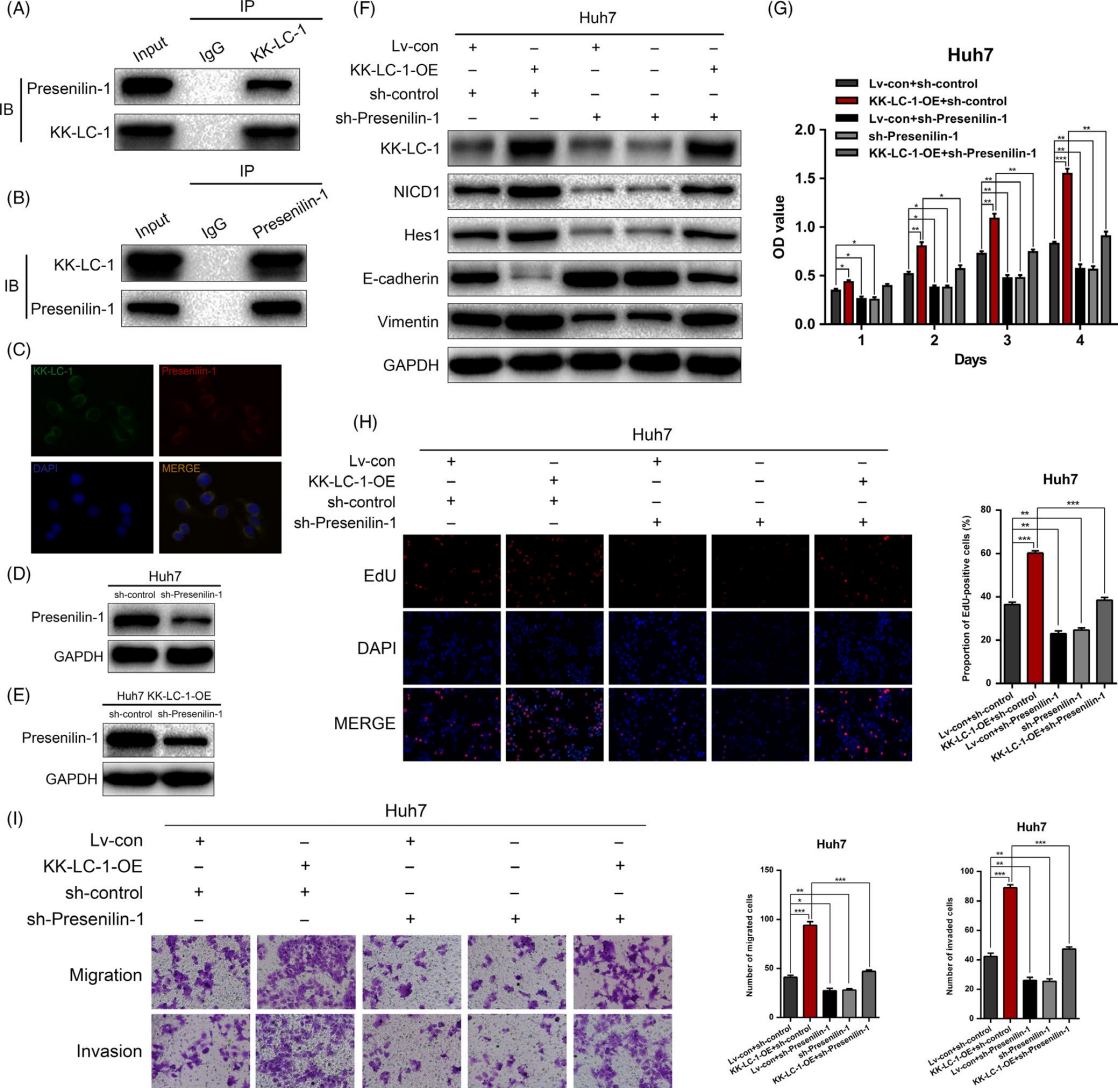
KK‐LC‐1 physically interacts with presenilin‐1 to regulate the Notch1 signalling in HCC. A, presenilin‐1 was detected in cell lysates immunoprecipitated with an anti‐KK‐LC‐1 antibody. B, KK‐LC‐1 was detected in cell lysates immunoprecipitated with an anti‐presenilin‐1 antibody. C, Double immunofluorescence staining of KK‐LC‐1 and presenilin‐1 was conducted to show that KK‐LC‐1 and presenilin‐1 colocalized with each other. Cell nuclei were stained with DAPI. D, presenilin‐1 was knocked down using shRNA in Huh7 cells. E, presenilin‐1 was knocked down using shRNA in Huh7 KK‐LC‐1‐OE cells. F, Western blot was performed to evaluate the expressions of NICD1, Hes1, E‐cadherin and vimentin in Huh7 KK‐LC‐1‐OE cells with presenilin‐1 knockdown. G, CCK‐8 assays for Huh7 KK‐LC‐1‐OE cells with presenilin‐1 knockdown. H, EdU assays were conducted to assess the proliferation ability of Huh7 KK‐LC‐1‐OE cells with presenilin‐1 knockdown. Proportion of EdU‐positive cells was determined and statistically analysed. I, Transwell assays were used to analyse HCC cell motility in Huh7 KK‐LC‐1‐OE cells with presenilin‐1 knockdown. Migrated and invaded cells were quantified and statistically analysed. The data are shown as the mean ± SE. *P < 0.05, **P < 0.01, ***P < 0.001

We investigated the mechanism by which KK‐LC‐1 regulated the Notch1 signalling. Presenilin‐1 is a catalytic subunit of the γ‐secretase complex, which is an endoprotease complex that catalyses the S3 cleavage of Notch1. As the core component of γ‐secretase, presenilin‐1 functions as the upstream modulator of Notch1 signalling in various physiological and pathophysiological conditions. Increased expression of presenilin‐1 has recently been correlated to HCC cell proliferation.^29^ Therefore, we hypothesized that presenilin‐1 may be involved in KK‐LC‐1‐mediated liver cancer progression. We first investigated whether KK‐LC‐1 could interact with presenilin‐1 in HCCLM3 cells. As shown in Figure 8A, presenilin‐1 was detected in cell lysates immunoprecipitated with an anti‐KK‐LC‐1 antibody. Reciprocally, KK‐LC‐1 was immunoprecipitated with an anti‐presenilin‐1 antibody (Figure 8B). Double immunofluorescence staining further suggested that both proteins were colocalized in the cytoplasm (Figure 8C). To further address the role of presenilin‐1 in KK‐LC‐1‐mediated HCC progression, we knocked down the expression of presenilin‐1 using shRNA in Huh7 cells (Figure 8D) and Huh7 KK‐LC‐OE cells (Figure 8E). We also showed that the KK‐LC‐1‐induced Notch1 activity and EMT were restored after presenilin‐1 knockdown (Figure 8F). Rescue experiments showed that the malignant phenotypes in KK‐LC‐1‐OE Huh7 cells were ameliorated by presenilin‐1 silencing, as observed using the CCK‐8, EdU and transwell assays (Figure 8G‐I). Taken together, these results demonstrated that KK‐LC‐1 promoted Notch1‐mediated HCC progression through physically interacting with presenilin‐1.

### 3.7 CpG hypomethylation induces expression of KK‐LC‐1 in HCC

**Figure 9:**
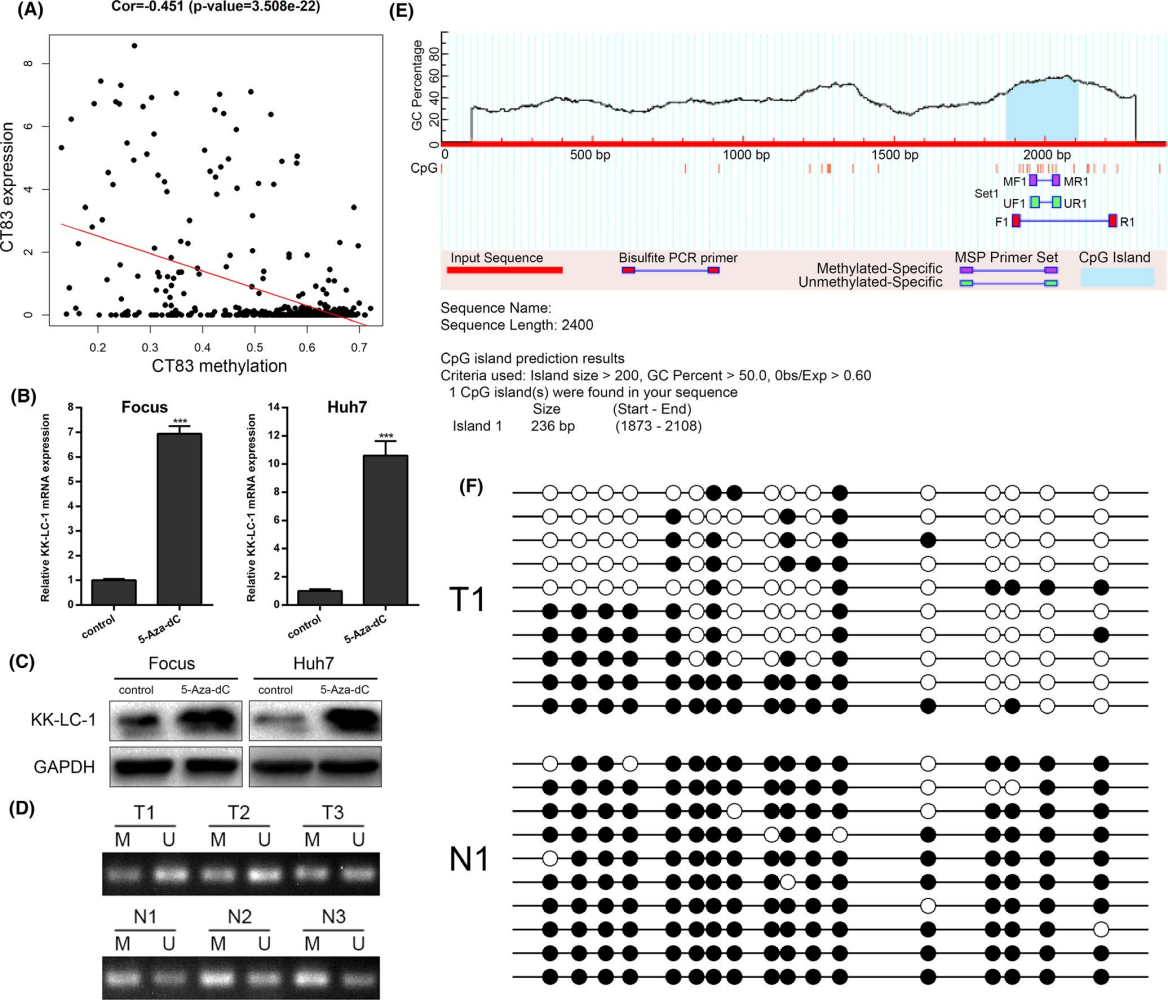
CpG hypomethylation induces expression of KK‐LC‐1 in HCC. A, GEO data were analysed to show the correlation between *CT83* DNA methylation levels and its expression levels in HCC (correlation coefficient = −0.451, *P* = 3.508 × 10^−22^). B, Focus and Huh7 cells were treated with DNA‐demethylating agent 5‐Aza‐dC, and the *CT83* mRNA expression levels were examined using RT‐qPCR. The data are shown as the mean ± SE. ***P < 0.001. C, Following 5‐Aza‐dC treatment, Western blot was performed to show KK‐LC‐1 protein expression level in Focus and Huh7 cells. D, *CT83* methylation in three paired HCC tissues (T) and non‐tumorous samples (N) was examined by methylation‐specific PCR. M: methylated; U: unmethylated. E, MethPrimer program was used to predict CpG islands of the *CT83* gene locus and design primers. F, Bisulphite sequencing PCR was performed to show the methylation status of *CT83* CpG islands using one matched HCC tissue and non‐tumorous specimen. Filled circles represent methylated CpG sites, and open circles represent unmethylated CpG sites.

Previous works suggested that *CT83*, the gene that encodes KK‐LC‐1 protein, is highly hypomethylated in the basal form of breast cancer.^6^, ^30^ Therefore, we wondered whether this epigenetic regulatory mechanism existed in HCC. We analysed two gene methylation microarrays (GSE37988 and GSE57956) and two gene expression microarrays (GSE14520 and GSE25097) from GEO database to explore the relationship between *CT83* DNA methylation levels and its expression levels in HCC. The results suggested a significant negative correlation between DNA methylation and expression level of *CT83* (Figure 9A; correlation coefficient = −0.451, *P* = 3.508 × 10^−22^). We then treated low KK‐LC‐1‐expressing HCC cell lines (Focus and Huh7) with 5 μmol/L DNA‐demethylating agent 5‐aza‐deoxycytidine (5‐Aza‐dC; Sigma‐Aldrich, St. Louis, MO, USA) for four days. Following 5‐Aza‐dC treatment, *CT83* expression was robustly upregulated in both of the low expressing cell lines (Figure 9B). Importantly, KK‐LC‐1 protein was also induced in these two cell lines treated with 5‐Aza‐dC (Figure 9C). To confirm the results in tissue samples, we performed MSP analysis on three paired HCC specimens (Figure 9D). The methylation rate of *CT83* in tumour tissues was markedly lower than that in the corresponding non‐tumorous tissues. We subsequently applied BSP analysis to examine the methylation status of the region upstream of *CT83* in one matched HCC tissue and non‐tumorous specimen (Figure 9E,F). According to the BSP results, we verified lower CpG methylation level in HCC tissue with higher expression level of *CT83* as compared to adjacent non‐tumorous liver tissue. Collectively, these data affirmed that the activation of KK‐LC‐1 in HCC is attributed to the CpG hypomethylation.

## 4 DISCUSSION

Cancer/testis antigens have emerged as a class of tumour‐associated antigens with promising clinical implications as biomarkers and immunotherapeutic targets for malignancies.^31^, ^32^ Many CT antigens have been revealed to be involved in liver cancer progression.^33^, ^34^ KK‐LC‐1, a CT antigen with remarkably higher expression rate than that of other CT antigens, possesses a great advantage in cancer targeted therapies.^10^ However, functional and mechanistic investigation of KK‐LC‐1 in human malignancies has never been reported before. In this study, we demonstrated for the first time that elevated KK‐LC‐1 played an important role in HCC progression. In vitro and in vivo experiments showed that KK‐LC‐1 knockdown dampened the proliferation, migration, invasion and EMT in HCC, whereas KK‐LC‐1 overexpression promoted HCC malignant phenotypes. Mechanistically, hypomethylation‐induced KK‐LC‐1 upregulation aggravated HCC via the Notch1/Hes1 signalling through binding to presenilin‐1.

Presenilin was first identified as the site of missense mutations leading to early‐onset familial Alzheimer's disease.^36^, ^37^ The association of presenilin‐1 with human malignancies has also been implicated.^38^, ^39^ A recent study has proposed the role of presenilin‐1 in facilitating HCC progression. As reported, presenilin‐1 level is elevated in HCC, accompanied by increased γ‐secretase activity. Presenilin‐1 promotes cell proliferation in HCC.^29^ Presenilin‐1‐dependent γ‐secretase is a crucial proteinase for Notch1 activation.^40^ γ‐secretase is a complex comprising Presenilin, anterior pharynx‐defective 1, nicastrin and Presenilin enhancer‐2. Presenilin is recognized as the catalytic subunit and is essential for the activity of γ‐secretase complex. Presenilin, together with other components, forms the functional enzyme to catalyse the processing of Notch.^41^ In the present study, we found that KK‐LC‐1 physically bound to presenilin‐1 and affected the downstream Notch signalling.

The Notch pathway is crucial for tissue and organ development and has been shown to participate in HCC progression.^42^, ^43^ In mammals, four types of Notch receptors (Notch1‐4) have been described. Among these receptors, Notch1 is dominantly expressed in hepatocytes.^44^ High Notch1 expression is associated with large tumour size, microvascular invasion and HCC metastasis.^45^ Notch1 overexpression predicts an unfavourable survival outcome for liver cancer patients.^46^ Our results showed that manipulation of KK‐LC‐1 was correlated with altered Notch activity. Inhibition of the Notch signalling by DAPT decreased the enhanced malignant phenotypes induced by KK‐LC‐1 overexpression, indicating that KK‐LC‐1 augmented HCC growth and metastasis through the Notch1 signalling.

Hypomethylated CpG islands have been reported to be associated with the activation of CT genes in several types of cancers. *CT45*, a 6‐member family of X‐linked CT genes, is upregulated in epithelial ovarian cancer. Elevated level of CT45 expression is demonstrated to be induced by promoter hypomethylation.^47^ In lung adenocarcinoma, *PIWIL1* has been identified as an extremely highly expressed CT gene. Promoter DNA hypomethylation of PIWIL1 causes its overexpression.^48^ In the present study, we found that hypomethylated CpG islands caused the upregulation of *CT83*, which is the gene that encodes KK‐LC‐1. We detected a lower rate of *CT83* methylation in HCC samples than that in the paired non‐tumorous tissues. As confirmed by BSP results, decreased CpG methylation level was associated with the reactivation of *CT83* in HCC. 5‐Aza‐dC treatment in HCC cells with low CT83 level led to elevated *CT83 *expression.

In conclusion, we demonstrated that hypomethylation‐induced KK‐LC‐1 facilitated HCC progression. KK‐LC‐1 was suggested as a prognostic indicator for HCC. Based on the mechanistic experiments, we found that KK‐LC‐1 physically interacted with presenilin‐1 to promote the Notch1/Hes1 pathway in HCC, contributing to a better understanding of the intricate regulatory network underlying HCC pathogenesis. Targeting the KK‐LC‐1/presenilin‐1/Notch1/Hes1 pathway could be a candidate for molecularly targeted therapy in HCC.

## CONFLICT OF INTEREST

The authors declare that they have no conflict of interests.

## AUTHORS’ CONTRIBUTIONS

ZQC, JDW and XHW contributed to study design; ZQC, XLZ, LYP, YZ, GYH, LZ, ZSW, WY, JJQ, XZD and HBS performed data collection and analysis; ZQC, XLZ, JDW and XHW contributed to manuscript preparation.
