# Supplementary material for: Hypomethylation‐mediated activation of cancer/testis antigen KK‐LC‐1 facilitates hepatocellular carcinoma progression through activating the Notch1/Hes1 signalling
Source: Cell Prolif. 2019 Mar 20;52(3):e12581. doi: 10.1111/cpr.12581 (PMC6536599; doi:10.1111/cpr.12581)
Supplement: Supplementary file 1 [file CPR-52-e12581-s001.docx]

| Gene | Forward primer | Reverse primer |
| --- | --- | --- |
| **RT-qPCR**^†^ |  |  |
| CT83 | CTCCTAGCGAGCAGCATTCTG | TTGATGACATTTCGCCAGTGT |
| GAPDH | AGCCACATCGCTCAGACAC | GCCCAATACGACCAAATCC |
| **Sequences for MSP**^‡^ |  |  |
| CT83 (Methylated) | TCGGTTTTTATATTATTTTATTGTGGC | TCTATATTATCCCGCTTTATATACGAC |
| CT83 (Unmethylated) | TGGTTTTTATATTATTTTATTGTGGTGT | AAATTCTATATTATCCCACTTTATATACAA |
| **Sequences for BSP**^§^ |  |  |
| CT83 | AAGGATTTGAAATTAATTAGGTAGG | CCAAAAAACAATCAAAACACACA |
| **Sequences for shRNA**^¶^ |  |  |
| sh1-KK-LC-1 | GCAGCATTCTGTGTGCCTTGA |  |
| sh2- KK-LC-1 | GGCGAAATGTCATCAAATTCA |  |
| sh3- KK-LC-1 | GCAATACAGACAACAATCTTG |  |
| sh-Presenilin-1 | GCTGTGGACTACATTACTGTT |  |

**TABLE S1** The list of primers and the sequences of shRNA

^†^*RT-qPCR* quantitative real-time PCR

^‡^*MSP* methylation specific PCR

^§^*BSP* bisulfite sequencing PCR

^¶^*shRNA* short hairpin RNA

**Table S2** Correlation between KK-LC-1 and clinicopathologic characteristics of patients with hepatocellular carcinoma

| Clinicopathologic characteristics | Total | KK-LC-1 expression | | *P* |
| --- | --- | --- | --- | --- |
|  |  | High (%) | Low (%) |  |
| Gender |  |  |  | 1.000 |
| Male | 45 | 22 (48.9) | 23 (51.1) |  |
| Female | 15 | 8 (53.3) | 7 (46.7) |  |
| Age |  |  |  | .119 |
| >60 | 27 | 10 (37.0) | 17 (63.0) |  |
| ≤60 | 33 | 20 (60.6) | 13 (39.4) |  |
| Cirrhosis |  |  |  | .671 |
| Presence | 54 | 28 (51.9) | 26 (48.1) |  |
| Absence | 6 | 2 (33.3) | 4 66.7) |  |
| HBV^†^ infection |  |  |  | .506 |
| Yes | 49 | 23 (46.9) | 26 (53.1) |  |
| No | 11 | 7 (63.6) | 4 (36.4) |  |
| α-fetoprotein (ng/ml) |  |  |  | .017* |
| > 400 | 24 | 17 (70.8) | 7 (29.2) |  |
| ≤ 400 | 36 | 13 (36.1) | 23 (63.9) |  |
| Edmondson-Steiner grading |  |  |  | .035* |
| III-IV | 25 | 17 (68.0) | 8 (32.0) |  |
| I-II | 35 | 13 (37.1) | 22 (62.9) |  |
| Tumor size |  |  |  | .606 |
| >5 cm | 29 | 13 (44.8) | 16 (55.2) |  |
| ≤5 cm | 31 | 17 (54.8) | 14 (45.2) |  |
| Microvascular invasion |  |  |  | .013* |
| Yes | 20 | 15 (75.0) | 5 (25.0) |  |
| No | 40 | 15 (37.5) | 25 (62.5) |  |
| Tumor multiplicity |  |  |  | .567 |
| Multiple | 17 | 10 (58.8) | 7 (41.2) |  |
| Single | 43 | 20 (46.5) | 23 (53.5) |  |
| TNM^‡^ stage |  |  |  | .003** |
| II-III | 17 | 14 (82.4) | 3 (17.6) |  |
| I | 43 | 16 (37.2) | 27 (62.8) |  |

**P* < .05; ***P* < .01.

^†^*HBV* hepatitis B virus

^‡^*TNM* tumor-node-metastasis

**Table S3** Univariate and multivariate analysis of the prognostic factors for overall survival and probability of recurrence

| Prognostic factors | Overall survival | | | | Probability of recurrence | | | |
| --- | --- | --- | --- | --- | --- | --- | --- | --- |
|  | Univariate Multivariate | | | | Univariate Multivariate | | | |
|  | Log-rank | *P* | HR^§^ (95% CI^¶^) | *P* | Log-rank | *P* | HR (95% CI) | *P* |
| Gender (Male/Female) | 1.240 | .266 |  |  | .916 | .339 |  |  |
| Age (>60/≤60) | .195 | .659 |  |  | .046 | .831 |  |  |
| Cirrhosis (Presence/Absence) | .142 | .706 |  |  | .017 | .897 |  |  |
| HBV^†^ infection (Yes/No) | 3.204 | .073 |  |  | 2.194 | .139 |  |  |
| α-fetoprotein (ng/ml) (>400 /≤ 400) | 10.706 | .001*** | Not included |  | 5.428 | .020* | 2.270 (1.023-5.035) | .044* |
| Edmondson-Steiner grading (III-IV/I-II) | 4.587 | .032* | 2.738 (1.180-6.353) | .019* | 3.668 | .055 |  |  |
| Tumor size (>5 cm/≤5 cm) | .771 | .380 |  |  | .082 | .774 |  |  |
| Microvascular invasion (Yes/No) | 7.847 | .005** | Not included |  | 13.898 | < .001*** | 2.483 (1.064-5.796) | .035* |
| Tumor multiplicity (Multiple/Single) | 1.538 | .215 |  |  | 2.357 | .125 |  |  |
| TNM^‡^ stage (II-III vs I) | 18.731 | < .001*** | 8.937 (3.402-23.475) | < .001*** | 18.458 | < .001*** | 3.293 (1.414-7.668) | .006** |
| KK-LC-1 expression (High/Low) | 9.765 | .002** | 5.912 (2.307-15.147) | < .001*** | 6.197 | .013* | 2.787 (1.232-6.305) | .014* |

**P* < .05; ***P* < .01; ****P* < .001.

^†^*HBV* hepatitis B virus

^‡^*TNM* tumor-node-metastasis

^§^*HR* hazard ratio

^¶^*CI* confidence interval
